# The complete chloroplast genome of *Stichorkis gibbosa* (Orchidaceae: Malaxideae)

**DOI:** 10.1080/23802359.2022.2111980

**Published:** 2022-08-29

**Authors:** Wen-Ting Yang, Kun-Lin Wu, Lin Fang, Tie-Long Wen, Song-Jun Zeng, Lin Li

**Affiliations:** a Key Laboratory of South China Agricultural Plant Molecular Analysis and Genetic Improvement, South China Botanical Garden, Chinese Academy of Sciences, Guangzhou, China; b Guangdong Provincial Key Laboratory of Applied Botany, South China Botanical Garden, Chinese Academy of Sciences, Guangzhou, China; c University of Chinese Academy of Sciences, Beijing, China

**Keywords:** Chloroplast genome, Orchidaceae, phylogeny, *Stichorkis gibbosa*

## Abstract

*Stichorkis gibbosa* is a rare orchid species of the tribe Malaxideae mainly distributed in tropical Asia. This is the only species of the genus *Stichorkis* Thouars which has been reported to occur in China. Despite the importance of this genus, previous molecular studies based on few markers have resulted in limited phylogenetic resolution. With the decline of habitats, the wild population of *S. gibbosa* has decreased in recent years. In this study, we first reported the complete chloroplast (cp) genome of *S. gibbosa*. The entire cp genome was determined to be 158,056 bp in length with overall GC content of 36.9%, containing a pair of inverted repeat regions (IRs) of 27,006 bp, separated by a large single-copy (LSC, 86,280 bp) and a small single-copy (SSC, 17,764 bp). A total of 133 unique genes were annotated, including 87 protein-coding genes, 38 tRNA genes, and eight rRNA genes. The phylogenetic tree indicated that *S. gibbosa* was a sister group of the genus *Oberonia* and the epiphytic *Liparis* alliance with strong support.

*Stichorkis* Thouars (du Petit Thouars 1809) of the tribe Malaxideae comprises approximately 60 species that are mainly distributed in tropical Asia including Borneo, Comoros, India, Jawa, Laos, Lesser Sunda Island, Malaya, Mauritius, Myanmar, Philippines, Réunion, Sri Lanka, Sulawesi, Sumatera, Thailand, and Vietnam (Naïve and Ormerod 2019). The genus *Stichorkis* had been relegated to the synonymy of *Liparis* Rich. *s.l.* for a long time, until it was resurrected to a separate genus based on molecular and morphological data and commonly recognized by various authors (Tang et al. 2015; Naïve and Ormerod 2019; Li et al. 2020). *Stichorkis* is composed of epiphytic or lithophytic species that inhabit humid montane forests in limestone hills. Members of this genus are characterized by having bilaterally flattened rachis, distichous and closely imbricate bracts, with commonly orange-red flowers. As the only representative of the genus *Stichorkis* known to occur in China, *Stichorkis gibbosa* (Fient) J.J. Wood 2011 is a rare orchid with a narrow distribution in Yunnan (Shui and Chen 2006; Chen et al. 2009; Wood et al. 2011). This species is easily recognizable by its distichous floral bracts and gibbous lip.

The chloroplast (cp) genome has been widely used in plant phylogenetics, evolutionary analyses, and population genetic studies. Several studies have found that plastomes provide unique advantages in resolving the phylogenetic relationships (Yang et al. 2013; Vieira et al. 2014). In this study, the complete cp genome sequence of *S. gibbosa* was first determined using next-generation sequencing (NGS). The result provided valuable genomic information for *S. gibbosa*, which would be beneficial for further phylogenetic studies on the related genera and species in the subtribe Malaxidinae (Chase et al. 2015).

Living material of *S. gibbosa* was first collected from Yunnan Province, China in 2020 and introduced to cultivation in the greenhouse of South China Botanical Garden, Chinese of Academy of Sciences (SCBG, CAS, 23°10.858′N, 113°21.136′E, 27 m). The voucher specimen was deposited at the herbarium of South China Botanical Garden (IBSC), under collection number of YWT005 (Wenting Yang, yangwenting@scbg.ac.cn). Total genomic DNA was extracted using the Trelief plant genomic DNA kit (Tsingke Biological Technology, Beijing, China). The DNA library construction and high-throughput sequencing was performed using the DNBseq platform at Beijing Genomics Institute (BGI) in Wuhan, China. The plastome genome sequences of *Liparis auriculata* (MN200365), *L. bootanensis* (MN627759), *L. nervosa* (MN641753), and *L. pingtaoi* (MN627758) were downloaded from GenBank as references to assemble *S. gibbosa* by using GetOrganelle pipeline (Jin et al. 2018). We used Geneious R9.0.2 (Kearse et al. 2012) to verify the accuracy of the assembly results. The plastid genome annotator (PGA) (Qu et al. 2019) was performed for genome annotation, coupled with manual corrections for putative start and stop codons and intron/exon boundaries. The complete cp genome sequence of *S. gibbosa* was deposited in GenBank under the accession no. OM759993.

The complete cp genome of *S. gibbosa* was 158,056 bp in length and presented a typical quadripartite structure of the large single-copy region (LSC) of 86,280 bp, a small single-copy region (SSC) of 17,764 bp, and two inverted repeat regions (IRs) of 27,006 bp. The genome encoded a total of 133 functional genes, including 87 protein-coding genes, 38 tRNA genes, and eight rRNA genes. The GC content of the whole cp genome was 36.9%. The IR regions had higher GC content (43.1%) than the LSC (34.4%) and SSC (29.7%) regions due to the presence of GC-rich rRNA genes.

To explore the phylogenetic position of *S. gibbosa* and its related taxa within the tribe Malaxideae, we performed a phylogenetic analysis based on 19 cp genomes from 18 representative species of five genera, including 18 accessions downloaded from GenBank and the newly sequenced plastome for *S. gibbosa.* Following the previous study (van den Berg et al. 2005), three representatives of genera *Epipactis*, *Neottia*, and *Cephalanthera* were employed as outgroups. We aligned the whole 22 cp genomes using MAFFT Alignment and manually adjusted wherever necessary by Geneious R9.0.2. The maximum-likelihood (ML) analysis was performed with IQ-TREE 1.6.12 (Nguyen et al. 2015) using 1000 bootstrap replicates under the best-fit model GTR + F+I + G4.

In accordance with previous studies of the tribe Malaxideae (Cameron 2005; Tang et al. 2015; Li et al. 2020) based on partial DNA markers, our result indicated that *Stichorkis* was strongly supported to be the sister group of a subclade comprising the epiphytic *Liparis* alliance and *Oberonia* with a bootstrap value of 100%. The epiphytic and terrestrial taxa of Malaxidinae were resolved into two major lineages (Figure 1). The complete plastid genome information of *S. gibbosa* reported in this paper provided data useful for population genomic studies, conservation works and the genetic diversity of *Stichorkis* as well as for phylogenetic studies within *Liparis s.l*. of the subtribe Malaxidinae. We would expect a better resolved and more robust phylogeny of Malaxideae in the future when a more comprehensive sampling is available.

**Figure 1.**
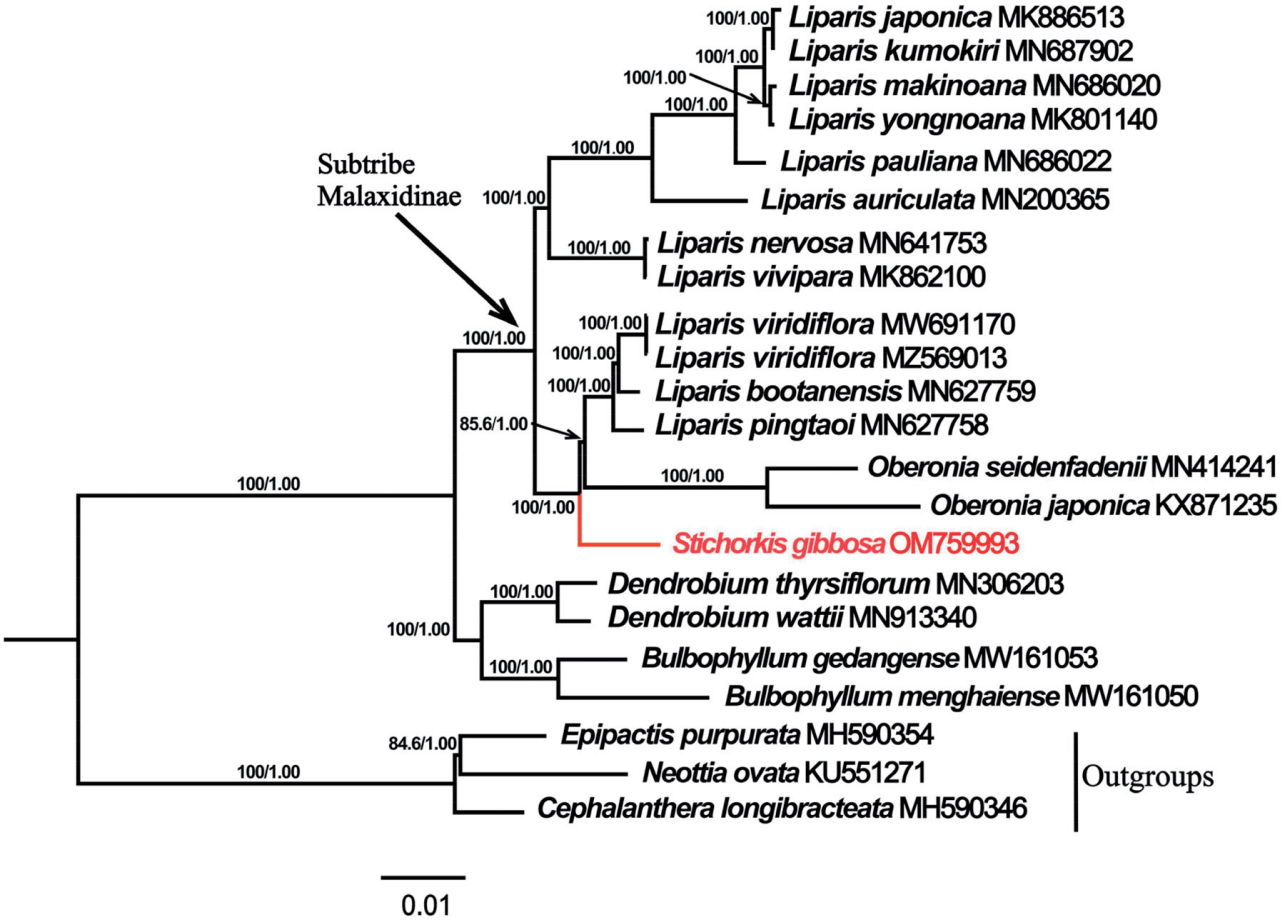
The maximum-likelihood (ML) phylogenetic tree of *Stichorkis* and its related genera in tribe Malaxideae based on complete chloroplast genome sequences, showing the position of *Stichorkis gibbosa* (in red). Numbers at nodes represent bootstrap support values (BP, left) and Bayesian’s posterior probabilities (PP, right), respectively.

## Data Availability

The complete plastid genome of *Stichorkis gibbosa* of this study is available in NCBI GenBank database (https://www.ncbi.nlm.nih.gov) with the accession number: OM759993. The associated BioProject, SRA, and BioSample numbers are PRJNA826671, SRR18750926, and SAMN27573741, respectively.
